# The Potentiality of Herbal Remedies in Primary Sclerosing Cholangitis: From *In Vitro* to Clinical Studies

**DOI:** 10.3389/fphar.2020.00813

**Published:** 2020-06-10

**Authors:** Elisa Ceccherini, Antonella Cecchettini, Maria Aurora Morales, Silvia Rocchiccioli

**Affiliations:** ^1^ Institute of Clinical Physiology, National Research Council (CNR), Pisa, Italy; ^2^ Department of Clinical and Experimental Medicine, University of Pisa, Pisa, Italy

**Keywords:** cholangiocytes, hepatic stellate cells, herbal, phytochemicals, primary sclerosing cholangitis, bioflavonoids

## Abstract

Primary sclerosing cholangitis is a complex pathological condition, characterized by chronic inflammation and fibrosis of the biliary epithelium. Without proper clinical management, progressive bile ducts and liver damage lead to cirrhosis and, ultimately, to liver failure. The known limited role of current drugs for treating this cholangiopathy has driven researchers to assess alternative therapeutic options. Some herbal remedies and their phytochemicals have shown anti-fibrotic properties in different experimental models of hepatic diseases and, occasionally, in clinical trials in primary sclerosing cholangitis patients; however their mechanism of action is not completely understood. This review briefly examines relevant studies focusing on the potential anti-fibrotic properties of *Silybum marianum*, *Curcuma longa*, *Salvia miltiorrhiza*, and quercetin. Each natural product is individually reviewed and the possible mechanisms of action discussed.

## Introduction

Primary Sclerosing Cholangitis (PSC) is a progressive cholestatic liver disease caused by chronic inflammation and fibrosis of the biliary epithelium, resulting in multi-focal bile duct strictures that affect intrahepatic and extrahepatic bile ducts, leading to cirrhosis and eventually hepatic failure (Levy and Lindor, 2006; Takakura et al., 2017). The clinical manifestations and complications related to PSC include abdominal pain, jaundice, infectious cholangitis, pruritus, vitamin deficiencies, metabolic bone disease, portal hypertension, varices, polyps, and malignancies, particularly cholangiocarcinoma (CCA) (Burak et al., 2004; Levy and Lindor, 2006; Razumilava et al., 2011; O'Toole et al., 2012; Franceschet et al., 2016; Karlsen et al., 2017; Takakura et al., 2017; Taghavi et al., 2018; Mertz et al., 2019). The etiology and pathogenesis of PSC remains elusive, although the key role of immune-mediated mechanisms is mostly accredited (Eaton et al., 2013; Tabibian et al., 2018). The exposure to environmental factors could trigger a complex interaction between adaptive and innate immune systems, leading to lymphocyte migration, cholangiocyte damage, and chronic fibrosis. Recently, genome-wide association studies have pointed out a strong correlation between PSC and genes able to regulate immune self-recognition and adaptive immunity (Karlsen et al., 2010; Melum et al., 2011; Ji et al., 2017). A “leaky gut” hypothesis has also been proposed, suggesting that the impairment of intestinal barrier function might lead to microbial translocation into bile (Navaneethan, 2015; Pontecorvi et al., 2016; Sabino et al., 2016; Tabibian et al., 2016; Giordano et al., 2018). PSC-associated cholangiocytes accumulate high level of bacterial lipopolysaccharides *in vivo* (Sasatomi et al., 1998); thus the impairment of gut-liver axis might trigger hepatobiliary inflammation and immune responses. The lack of unique and well-characterized pathogenesis still makes difficult the development of effective pharmacological therapies, which are actually aimed at treating symptoms and managing complications (Eaton et al., 2013; Lindor et al., 2015; Suri et al., 2019). Hence, liver transplantation remains the treatment of choice for end-stage PSC; however, recurrence occurs in the 20% to 40% of transplanted patients (Visseren and Darwish Murad, 2017).

In PSC, the concentric accumulation of connective tissue around intrahepatic and extrahepatic bile ducts (known as “onion-like” fibrosis) suggests that cholangiocytes play a central role in driving the fibrotic machinery (Eaton et al., 2013; O'Hara et al., 2017; Banales et al., 2019). Cholangiocytes are epithelial cells of the biliary tract that are quiescent in physiological condition. In chronic cholestatic liver disease, including PSC, cholangiocytes play a double role, being target, but also active subjects of the pathological status through multiple molecular processes. In response to liver injury, cholangiocytes acquire a reactive status which is characterized by increased expression of anti-apoptotic genes (Bcl-2) and adhesion molecules, and loss of some epithelial markers such as CK-7, CK-19, or E-cadherin in favor to functional and morphologic markers associated with mesenchymal phenotype (Kalluri and Neilson, 2003; Omenetti et al., 2008; Rygiel et al., 2008; Fabris et al., 2016). Once activated, cholangiocytes release numerous chemokines, cytokines, growth factors, neuroendocrine molecules, and other proinflammatory and fibrogenic mediators that act in autocrine and paracrine manners, activating other cell types (hepatic stellate cells (HSCs), portal fibroblasts, myofibroblasts, and hepatocytes) and recruiting immune cells that promote biliary damage (Lazaridis et al., 2004; Fabris et al., 2016; Fabris et al., 2017; Banales et al., 2019). *In vitro* and *in vivo* studies have partially highlighted the importance of cell-cell communication between cholangiocytes and HSCs, which involves multiple signaling pathways related to transforming growth factor-beta 1 (TGF-β1), Smad, hepatocyte nuclear factor 3-β, and integrin αvβ/nuclear factor-kappa B (NFkβ) axis (O'Hara et al., 2013; Kim et al., 2015; McDaniel et al., 2017). Indeed, activated HSCs seem to represent the main contributors to fibrosis in cholestatic liver disease, including PSC (Fickert et al., 2014). Under healthy condition, HSCs exert an equilibrium between extracellular matrix production and degradation; however, following activation especially *via* TGF-β1, HSCs show a proliferative status, loss of vitamin A and lipid droplets, increased expression of alpha-smooth muscle actin (α-SMA), and synthesis of extracellular matrix components (De Minicis et al., 2007; Mallat and Lotersztajn, 2013). The role of matrix metalloproteinases (MMPs) and tissue inhibitors of metalloproteinases (TIMPs) is well documented in regulating the levels of extracellular matrix (Visse and Nagase, 2003). Interestingly, decreased activity of MMPs is reported in liver fibrosis as a consequence of TIMPs overexpression in activated HSCs (Arthur et al., 1998). Briefly, the fibrotic process requires the involvement of different cells and inflammatory and pro-fibrogenic mediators. It is worth noticing that these mediators are mainly modulated through redox-sensitive reactions (Poli, 2000; Sánchez-Valle et al., 2012; Luangmonkong et al., 2018). Despite the efforts of scientists in testing the efficacy of new and old molecules, the major challenge in PSC remains the chronic progression of hepatobiliary fibrosis towards liver failure.

A wide range of herbal remedies has shown promising effects against hepatic fibrosis either in experimental models or even in preliminary clinical trials (Latief and Ahmad, 2017). In this review, we discuss four plant-derived products that have shown anti-fibrotic properties in multiple mouse models of hepatic fibrosis and in few clinical trials in PSC patients.

## Natural Products Active as Hepatic Antifibrotic Agents

A Medline search was performed in order to identify relevant published reports. The key words “herbal,” “phytotherapy,” “phytochemicals,” “bioflavonoids,” “plant extracts” were cross-referenced with “primary sclerosing cholangitis” and “hepatic fibrosis.” Among the many herbal remedies and phytochemicals that exert antifibrotic properties in different hepatic diseases, we focused our attention on four of them. In particular, *Silybum marianum* and *Curcuma longa* have been investigated in clinical trials in PSC patients; *Salvia miltiorrhiza* exerts antifibrotic properties in different chemically-induced liver fibrosis mouse models; and quercetin is a flavonoid presents in silymarin mixture and also it is daily taken with the diet.

### *Silybum marianum*

*Silybum marianum*, also known as milk thistle, has been used since long time in the management of liver diseases and biliary disorders. Silymarin is a mixture of flavonolignans containing silybins A and B, isosilybins A and B, silychristin, silydianin, and in smaller quantities, flavonols 2.3 dehydrosilybin, quercetin, (+) taxifolin, and kaempferol (Calani et al., 2012). The safety and the efficacy of silymarin have been investigated in PSC patients in an open-label pilot study (Angulo et al., 2008). A significant reduction in ALP and AST occurred following oral treatment with silymarin (140 mg three times daily for one year). No statistically significant changes in serum bilirubin and albumin levels were registered, indicating a possible arrest of PSC progression during silymarin treatment. The immunomodulatory, antioxidant, and antifibrotic effects of this natural mixture have been also investigated in several experimental models of liver fibrosis (Boigk et al., 1997; Tsai et al., 2008; Clichici et al., 2016; Federico et al., 2017; Karimi et al., 2018). In carbon tetrachloride–induced liver fibrosis in rats, silymarin significantly decreased the level of AST, ALT, and ALP in serum, and inhibited the increased expressions of α-SMA in liver tissue (Tsai et al., 2008). The α-SMA is a well-established marker of HSCs activation, and the reduction of this protein levels is related to the inhibition of activated HSCs (Carpino et al., 2005; De Minicis et al., 2007; Mallat and Lotersztajn, 2013). Interestingly, Clichici and colleagues confirmed the effectiveness of silymarin as an antifibrotic agent in carbon tetrachloride–treated mice through the reduction of α-SMA and TGF-β1 expression (Clichici et al., 2015; Clichici et al., 2016). In thioacetamide-induced chronic liver fibrosis, silymarin also demonstrated antifibrotic properties (Chen et al., 2012). The antifibrotic effects were primarily attributed to reduced hepatic levels of TIMP-1/2, whose overexpression is related to HSCs activation, TGF-β1, α-SMA, and collagen I expression (Kara et al., 2008; Chen et al., 2012). These data suggest that silymarin could exert its antifibrotic properties on cholangiocytes/HSCs axis perhaps inhibiting HSCs activation, which represents a key event in PSC development (Fickert et al., 2014). Moreover, *Silybum marianum* is well known for its antioxidant and anti-inflammatory properties on multiple hepatic disorders *via* the modulation of various transcription factors (Loguercio et al., 2007; Post-White et al., 2007; Federico et al., 2008; Trappoliere et al., 2009; Loguercio and Festi, 2011; Loguercio et al., 2012; Stiuso et al., 2014).

### *Salvia miltiorrhiza*

*Salvia miltiorrhiza* is a very popular traditional Chinese remedy widely used to treat different pathological conditions, including cardiovascular diseases, tumors, and cerebrovascular diseases (Yang et al., 2010; Zhou et al., 2011; Wang W.H. et al., 2017; Wang L. et al., 2017). This herbal medicine contains multiple lipophilic compounds and hydrophilic phenolic acids (Zhang et al., 2012; Cai et al., 2016).

*Salvia miltiorrhiza* has been shown to attenuate liver fibrosis in multiple experimental models (Wasser et al., 1998; Parajuli et al., 2015; Peng et al., 2018). *Salvia miltiorrhiza* is able to reverse hepatic fibrosis in rats following exposition to carbon tetrachloride, lowering levels of TGF-β1, procollagens I and III (Wasser et al., 1998). As previously reported, TGF-β1 represents the main activator of HSCs, which are responsible of the synthesis of extracellular matrix components (De Minicis et al., 2007; Mallat and Lotersztajn, 2013). A recent study demonstrated that the antifibrotic effect of S*alvia miltiorrhiza* is correlated with the increased activity of hepatic natural killers (NK) and inhibition of HSCs activation, as confirmed by diminished levels of α-SMA (Peng et al., 2018). Accumulating evidences suggest that NK cells play a pivotal role in controlling liver fibrosis through killing activated HSCs, as reported in both human and animal experiments (Muhanna et al., 2011; Fasbender et al., 2016; Shi et al., 2017). The anti-fibrotic effect of NK cells is suppressed during advanced liver injury, contributing to the progression of liver fibrosis (Jeong et al., 2011). Therefore, the ability of *Salvia miltiorrhiza* in restoring and promoting the activities of NK cells might represent an important anti-fibrotic mechanism. Most interesting, the immunomodulatory activity of *Salvia miltiorrhiza* was also demonstrated in BALB/c mice following *Listeria monocytogenes* infection, as confirmed by the increased number of peripheral monocytes and NK cells (Gao et al., 2012). It is interesting to remind the antifibrotic effects of PF2401-SF, a standardized and purified fraction of *Salvia miltiorrhiza*, in thioacetamide and carbon tetrachloride-induced liver fibrosis in rats (Parajuli et al., 2013; Parajuli et al., 2015). In these chemically-induced hepatic fibrosis models, *Salvia miltiorrhiza* seems to exert its action on HSC activation that might be mediated by downregulation of pivotal markers of fibrosis, including α-SMA, collagen I, and TIMP1. It is also well known that *Salvia miltiorrhiza* protects liver attenuating inflammatory reactions (Xie et al., 2014; Ma et al., 2016) and exerting antioxidant effects (Zhang et al., 1990; Zhao et al., 2006).

### *Curcuma longa*

*Curcuma longa* (Turmeric) has been used for centuries in both Ayurvedic and Chinese medicine for its anti-inflammatory properties in a wide repertoire of pathological conditions (Pari et al., 2008). Turmeric contains primarily curcumin, a phenolic compound, and three different analogs of curcumin (diferuloylmethane, demethoxycurcumin, and bisdemethoxycurcumin), as well as resins and volatile oils (tumerone, atlantone, and zingiberone) (Jurenka, 2009). More than 50 clinical studies assessed or are currently evaluating the pharmacological effects of curcumin in many different disorders in man. Recently, an open-label pilot study conducted at the Mayo Clinic, was focused on the safety and effectiveness of oral administration (750 mg twice a day for 12 weeks) of BCM-95 CG, a novel bioenhanced preparation of curcumin, in PSC patients (Eaton et al., 2019). Despite the low number of enrolled patients, a large amount of preclinical data suggest that antifibrotic properties of curcumin are relevant in chronic hepatic fibrosis. In particular, Baghdasaryan and colleagues highlighted the ability of curcumin in reducing bile duct injury and biliary fibrosis in Mdr2−/− mice (Baghdasaryan et al., 2010), which are currently used as a murine model for sclerosing cholangitis (Fickert et al., 2004). During cholangiopathies, activated cholangiocytes upregulate the expression of adhesion molecules and proinflammatory mediators responsible of the recruitment of immune cells (Lazaridis et al., 2004; Fabris et al., 2016; Fabris et al., 2017; Banales et al., 2019). In this respect, increased expression of vascular cell adhesion molecule 1 (VCAM-1) by cholangiocytes contributes to the persistence of liver inflammation through the recruitment of monocytes and lymphocytes, and mediating leukocyte adhesion by binding α4β1 integrins (Afford et al., 2014). Interestingly, curcumin is able to reduce VCAM-1 protein levels in Mdr2−/− mice *via* peroxisome proliferator-activated receptor gamma (PPARγ) signaling, without affecting the expression of stimulatory pro-inflammatory cytokines (Baghdasaryan et al., 2010). These results are consistent with the reduction of bile duct proliferation and biliary fibrosis observed following treatment with PPARγ agonists in bile duct-ligated (BDL) mouse models (Marra et al., 2000; Marra et al., 2005). Thus, the antifibrotic properties of curcumin could be mediated, in part, by the activation of PPARγ in activated cholangiocytes. Other studies have also demonstrated the key role of PPARγ expression in the maintenance of the quiescent HSCs phenotype (Hazra et al., 2004a; Hazra et al., 2004b). Consistently, the depletion of PPARγ together with the increased activation of nuclear factor NF-κB and ERK have been reported in *in vitro* activated human and rat HSCs (Elsharkawy et al., 1999; Hazra et al., 2004b; Foglia et al., 2019). Curcumin inhibited HSCs activation *in vitro* through the increased PPARγ expression and stimulation of PPARγ signaling, resulting in the inhibition of NF-κB activity (Xu et al., 2003; Zheng and Chen, 2004; Chen and Zheng, 2008). Furthermore, curcumin suppressed the expression of connective tissue growth factor in activated HSCs *in vitro* through NF‐κB inhibition, leading to the reduction of extracellular matrix components synthesis, including collagen I (Zheng and Chen, 2006; Chen and Zheng, 2008). Moreover, curcumin inhibits ERK signaling pathway, whose involvement in the activation of HSCs has already been demonstrated (Chen and Zheng, 2008; Foglia et al., 2019). Accumulating evidences indicate curcumin as a compound that possesses anti-inflammatory (Salama et al., 2013; He et al., 2015; Lee et al., 2020) ref) and protective effects towards oxidative associated liver diseases (Reyes-Gordillo et al., 2007; Fu et al., 2008; Salama et al., 2013; Liu et al., 2016; Samarghandian et al., 2017).

### Quercetin

Quercetin is a natural flavonoid widely present in many vegetables and fruits belonging to the family of *Apiaceae, Brassicaceae, Rosaceae* (Anand David et al., 2016; Babaei et al., 2018). It possesses various hepatoprotective properties, including antioxidant, antiviral, anti-inflammatory, anti-proliferative, and antifibrotic effects (Russo et al., 2012; Li et al., 2016; Caddeo et al., 2019; Lesjak et al., 2018; Xu et al., 2019). The positive effect of quercetin on liver fibrosis has been demonstrated both *in vitro* and in several murine models (Pavanato et al., 2003; Marcolin et al., 2012; Casas-Grajales et al., 2017; Wang R. et al., 2017; Wu et al., 2017; Li et al., 2018). In an *in vivo* study, Wu and colleagues observed inhibition of HSCs activation and proliferation following treatment with quercetin (Wu et al., 2017). These data are consistent with other *in vivo* studies that reported the anti-hepatofibrotic properties of quercetin in chemically-induced liver fibrosis mouse models (Pavanato et al., 2003; Casas-Grajales et al., 2017; Wang R. et al., 2017; Li et al., 2018). Quercetin exhibited anti-fibrogenic activity by regulation of HSCs-activation markers (e.g. α-SMA, Collagen I, TIMP-1) (Hernández-Ortega et al., 2012; Wang R. et al., 2017). Indeed, quercetin inhibited the activation of NF-κB in a dose-dependent manner *via* inhibition of IкBα degradation and decreased the expression of p38 MAPK by inhibiting its phosphorylation. These data are consistent with the well-documented role of p38MAPK/NF-κB axis in inflammation and HSCs activation (Schnabl et al., 2001; Luedde and Schwabe, 2011; Taniguchi and Karin, 2018; Czauderna et al., 2019). Novel evidences into quercetin activity have shown the inhibition of liver fibrosis through the regulation of macrophage activation and function *via* Notch1 pathway in carbon tetrachloride-treated mice (Li et al., 2018). Macrophage Notch1 expression was increased during liver injury in mice but quercetin treatment reversed this effect. These results are consistent with the role of Notch pathway in macrophage-mediated inflammation (Xu et al., 2015a; Eun and Jeong, 2016; Kimball et al., 2017), including certain hepatic diseases (Geisler and Strazzabosco, 2015; Xu et al., 2015b). Several studies indicate that autophagy may represent a key role for the modulation of numerous signaling pathways related to HSC activation (Thoen et al., 2011; Hernández-Gea et al., 2012). Thoen and colleagues have demonstrated that HSC activation is followed by an increased autophagic flux whose inhibition can in part inhibit HSCs (Thoen et al., 2011; Thoen et al., 2012). Moreover, quercetin was demonstrated to ameliorate liver fibrosis reducing HSCs autophagy through the regulation of the TGF-β1/Smad axis (Wu et al., 2017). It is interesting to note that this signaling pathway is related to the modulation of extracellular matrix gene expression causing fibrosis (Das et al., 2014).

## Conclusions

**Figure 1:**
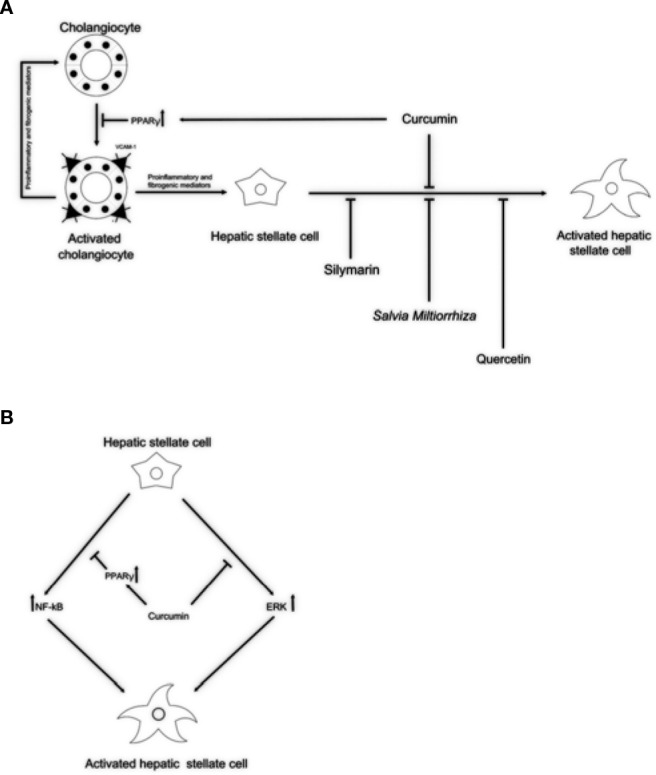
Potential anti-fibrotic properties of natural remedies in cholangiopathies. **(A)** By activation of PPARγ, curcumin might block reactive cholangiocytes and their inflammatory activation through the reduction of VCAM-1 expression, resulting in decreased attraction of immune cells. Silymarin, *Salvia miltiorrhiza,* and quercetin might exert its antifibrotic properties acting directly on HSCs activation. **(B)** In addition, acting as PPARγ activator and by inhibiting the ERK signaling curcumin might block HSCs activation. This dual role of curcumin could explain the antifibrotic properties exerted in experimental models and clinical trials. PPARγ, peroxisome proliferator-activated receptor gamma; VCAM-1, vascular cell adhesion molecule 1; ERK, extracellular signal–regulated kinases; HSCs, hepatic stellate cells.

Currently, effective therapies in arresting hepatobiliary fibrosis and restoring proper liver function in PSC are lacking; therefore, alternative therapies, which could help in the clinical management of PSC have been approached. Some herbs and phytochemicals have shown anti-fibrotic properties in different experimental models of liver fibrosis and also in PSC patients. **Table 1** is a summary of the literature exploring pharmacological effects and cellular targets of the herbal remedies taken into account in this review. Briefly, silymarin, curcumin, *Salvia miltiorrhiza,* and quercetin have been demonstrated to inhibit stellate cell activation (**Figure 1A**). Curcumin has a dual role, affecting both PPARγ and ERK signaling blocking HSC activation. In addition, through PPARy, it also affects cholangiocytes (**Figure 1B**). The ability of these natural products in acting on cholangiocytes/HSCs axis could represent an encouraging antifibrotic therapeutic opportunity. Moreover, the plant-derived phytoconstituents have antinflammatory and anti-oxidant activity that could impact positively on biliary cholestasis and fibrosis. The evidences supporting the use of herbs and phytochemicals in the management of PSC are insufficient; crude herbs and phytoproducts need to be examined also for their potential toxic effects. Nevertheless, due to the complexity of PSC and the lack of established cures, the roles of *Silybum marianum*, *Curcuma longa*, *Salvia miltiorrhiza,* and quercetin should be better evaluated in properly designed clinical studies. The possible use of these natural compounds alone or in combination with traditional drugs could represent a very promising field for future researches and therapies.

**Table 1:** Summary of references on pharmacological effects and cellular targets of the herbal remedies *Silybum marianum*, *Salvia miltiorrhiza*, *Curcuma longa,* and quercetin discussed in this review.

	Pharmacological effects				Cellular targets		
	Antifibrotic	Anti-inflammatory	Anti-oxidant	Immunomodulatory	Cholangiocyte	HSC	NK
*Silybum marianum*	Boigk et al., 1997; Tsai et al., 2008; Chen et al., 2012; Clichici et al., 2016; Federico et al., 2017	Federico et al., 2017	Federico et al., 2017	Karimi et al., 2018		Kara et al., 2008; Chen et al., 2012; Clichici et al., 2015; Clichici et al., 2016	
*Salvia miltiorrhiza*	Wasser et al., 1998; Parajuli et al., 2013; Parajuli et al., 2015; Peng et al., 2018	Xie et al., 2014; Ma et al., 2016	Zhang et al., 1990; Zhao et al., 2006	Gao et al., 2012; Peng et al., 2018		Peng et al., 2018	Peng et al., 2018
*Curcuma longa*	Baghdasaryan et al., 2010	Salama et al., 2013; Lee et al., 2020	Reyes-Gordillo et al., 2007; Fu et al., 2008; Salama et al., 2013; Liu et al., 2016; Samarghandian et al., 2017		Marra et al., 2000; Marra et al., 2005	Zheng and Chen, 2006; Chen and Zheng, 2008; Foglia et al., 2019	
Quercetin	Pavanato et al., 2003; Casas-Grajales et al., 2017; Wang R. et al., 2017; Wu et al., 2017; Li et al., 2018	Li et al., 2016; Lesjak et al., 2018	Lesjak et al., 2018; Caddeo et al., 2019; Xu et al., 2019	Li et al., 2016; Li et al., 2018		Schnabl et al., 2001; Luedde and Schwabe, 2011; Hernández-Ortega et al., 2012; Wang R. et al., 2017; Wu et al., 2017; Taniguchi and Karin, 2018; Czauderna et al., 2019	Li et al., 2018

## Author Contributions

All authors contributed to reviewing the current literature and writing of the manuscript and approved the final version of the paper. Conceptualization: EC, MM, SR. Original draft preparation: EC, AC. Final editing: EC, AC, MM, SR.

## Funding

The authors gratefully acknowledge the financial support ofAIRCS-Italian Association for Sclerosing Cholangitis Research.

## Conflict of Interest

The authors declare that the research was conducted in the absence of any commercial or financial relationships that could be construed as a potential conflict of interest.
